# A virtual reality home-based training for the management of stress and anxiety among healthcare workers during the COVID-19 pandemic: study protocol for a randomized controlled trial

**DOI:** 10.1186/s13063-022-06337-2

**Published:** 2022-06-02

**Authors:** Federica Pallavicini, Eleonora Orena, Simona di Santo, Luca Greci, Chiara Caragnano, Paolo Ranieri, Costanza Vuolato, Alessandro Pepe, Guido Veronese, Stefano Stefanini, Federica Achille, Antonios Dakanalis, Luca Bernardelli, Francesca Sforza, Angelo Rossini, Carlo Caltagirone, Sara Fascendini, Massimo Clerici, Giuseppe Riva, Fabrizia Mantovani

**Affiliations:** 1 Department of Human Sciences for Education, University of Milano Bicocca, Riccardo Massa”, Milan, Italy; 2 Foundation IRCCS, Neurological Institute Carlo Besta, Milan, Italy; 3 IRCCS Fondazione Santa Lucia, Rome, Italy; 4 Università Degli Studi Di Roma Tor Vergata, Rome, Italy; 5 Institute of Intelligent Industrial Technologies and Systems for Advanced Manufacturing (STIIMA), National Research Council of Italy (CNR), Lecco, Italy; 6 University of Milano Bicocca, Department of Psychology, Milan, Italy; 7 University of Milano-Bicocca, Specialization School in Psychology, Lecco, Italy; 8 Fondazione Europea Ricerca Biomedica (FERB), Gazzaniga, Italy; 9 Department of Medicine and Surgery, University of Milano Bicocca, Monza, Italy; 10 Become-Hub, Milan, Italy; 11 Humane Technology Lab, Università Cattolica del Sacro Cuore, Milan, Italy; 12 Applied Technology for Neuro-Psychology Lab, Istituto Auxologico Italiano, Milan, Italy

**Keywords:** Virtual reality, Stress, Anxiety, Psychoeducation, Relaxation, Healthcare workers, COVID-19

## Abstract

**Background:**

Healthcare workers represent one of the most affected categories by the adverse effects of the COVID-19 pandemic on mental health. Excessive stress and anxiety are critical factors that could compromise work performance. Besides, high levels of stress and anxiety may have long-term physical and psychological consequences. Recent studies investigated virtual reality to reduce stress and anxiety among healthcare workers during the COVID-19 pandemic. However, the proposed virtual reality interventions have important limitations related to their location (i.e., research lab and hospitals) and content (i.e., virtual experiences only for relaxation). Within this context, this randomized controlled trial aims to investigate the efficacy and acceptability of a brief home-based virtual reality training for managing stress and anxiety during the COVID-19 crisis in a sample of Italian healthcare workers.

**Methods:**

The study is a randomized controlled trial. It includes two groups of 30 individuals recruited from healthcare workers: (1) the experimental group and (2) the control group. Participants in the experimental group will receive a training consisting of three home sessions performed in a week. In each session, participants will try through an immersive virtual reality standalone system (i.e., Oculus Quest 2) a virtual psychoeducation experience on stress and anxiety (i.e., MIND-VR). Subsequently, they will try the virtual relaxation content (i.e., The Secret Garden). The control group will receive no training and will be reassessed one week and one month after the initial evaluation.

**Discussion:**

If the proposed brief home-based virtual reality training will result helpful and easy to use, it could become an empirically assessed viable option for protecting healthcare workers’ mental health both during the COVID-19 pandemic and once it will be over. Furthermore, the intervention might be easily adapted for other categories of people who need support in managing stress and anxiety.

**Trial registration:**

ClinicalTrials.gov NCT04611399.

## Administrative information

**Table:** 

Title {1}	A Virtual Reality Home-Based Training for the Management of Stress and Anxiety Among Healthcare Workers During the COVID-19 Pandemic: Study Protocol for a Randomized Controlled Trial
Trial registration {2a and 2b}.	NCT04611399 (ClinicalTrials.gov)
Protocol version {3}	05/03/22 Version 3.0
Funding {4}	The authors received no specific funding for this work.
Author details {5a}	Federica Pallavicini^1^*, Eleonora Orena^2^, Simona di Santo^3, 4^, Luca Greci^5^, Chiara Caragnano^6^, Paolo Ranieri^7^, Costanza Vuolato^2^, Alessandro Pepe^1^, Guido Veronese^1^, Stefano Stefanini^8^, Federica Achille^8^, Antonios Dakanalis^9^, Luca Bernardelli^10^, Francesca Sforza^10^, Angelo Rossini^3^, Carlo Caltagirone^3^, Sara Fascendini^8^, Massimo Clerici^9^, Giuseppe Riva^11,12^, Fabrizia Mantovani^1^ 1 University of Milano Bicocca, Department of Human Sciences for Education “Riccardo Massa”, Milan (Italy) 2 Foundation IRCCS Neurological Institute Carlo Besta, Milan (Italy) 3 IRCCS Fondazione Santa Lucia, Roma (Italy) 4 Università degli Studi di Roma Tor Vergata, Roma (Italy) 5 Institute of Intelligent Industrial Technologies and Systems for Advanced Manufacturing (STIIMA) National Research Council of Italy (CNR), Lecco (Italy) 6 University of Milano Bicocca, Department of Psychology, Milano (Italy) 7 University of Milano Bicocca, Specialization School in Psychology, Milan (Italy) 8 Fondazione Europea Ricerca Biomedica (FERB), Gazzaniga (Italy) 9 University of Milano Bicocca, Department of Medicine and Surgery, Monza (Italy) 10 Become-Hub, Milan (Italy) 11 Humane Technology Lab., Università Cattolica del Sacro Cuore, Milan (Italy) 12 Applied Technology for Neuro-Psychology Lab., Istituto Auxologico Italiano, Milan (Italy)
Name and contact information for the trial sponsor {5b}	The authors received no specific funding for this work.
Role of sponsor {5c}	The authors received no specific funding for this work.

## Introduction

### Background and rationale

After the first reports at the end of December 2019 of unidentified pneumonia cases in Wuhan, China, on March 11, 2020, the World Health Organization (WHO) declared the novel coronavirus (COVID-19) a global pandemic [1]. Healthcare workers represent one of the most affected categories by the adverse effects of the COVID-19 crisis [2–4]. The risk of being infected, exhausting work rhythms, and the need to manage patients experiencing extreme suffering have put hospital staff’s physical and mental health at high risk [2–4].

Several studies and systematic reviews showed that healthcare workers, especially those working in intensive care units (ICU), emergency medicine, infectious disease, and pulmonary medicine, have experienced high levels of stress [4, 5] and anxiety [6, 7] during the outbreak of the COVID-19 pandemic. In Italy, among the first European countries to be hit by the COVID-19 pandemic, healthcare workers showed high levels of stress, anxiety, and depression and an increased risk for post-traumatic stress disorder (PTSD) [8–10]. Similar results have been reported by studies conducted in several countries around the world, including China [11, 12], the USA [13, 14], and India [15, 16].

Excessive stress and anxiety are critical factors that could compromise healthcare workers’ performance [17, 18], particularly during an emergency [19]. Besides, high stress and anxiety levels may have long-term physical and psychological consequences [20, 21]. Therefore, after the COVID-19 outbreak more than ever, urgent actions are needed to offer healthcare workers psychological support [22, 23].

Within this context, new approaches to mental health integrating advanced technologies such as virtual reality (VR) can play a critical role [24–29]. From a technical point of view, VR is a set of technology, including head-mounted display (HMD), computer, and mobile devices, that lets users navigate and interact in real time with a three-dimensional (3D) environment [30, 31]. VR technologies can immerse their users in a virtual environment to different degrees [32]: from a simple presentation on a two-dimensional (2D) display screen system (i.e., desktop VR) to a room-size system (i.e., semi-immersive VR), often referred to by the trade name of C-Automatic Virtual Environment (CAVE), up to highly immersive systems (i.e., immersive VR), that used HMDs. The HMD, a device containing two LCD screens capable of tracking the user’s head motions and position to portray the virtual environments accordingly, is considered the unquestionable leader of immersive VR systems [33]. Such systems can be classified based on the technologies on which they are implemented (Table 1).

**Table 1:** Classification of immersive VR systems based on the technologies on which it is implemented

Typology	Definition	Examples
PC-based	Require a connection between an HMD and a computer with advanced computational and graphics capabilities	Oculus Rift S, HTC Vive
Console-based	Need a connection between an HMD and a gaming console	PlayStation VR
Mobile	Consist in the integration of VR on mobile devices thanks to specific HMDs	Low-cost HMDs compatible with mobile phones such as Google Cardboard
Standalone (all-in-one)	They do not need other technologies to work	Oculus Quest 2, HTC Vive Focus, Pico Interactive Neo

VR has been successfully applied in a wide range of mental conditions [34–37], including stress and anxiety [38–41]. VR is effective in inducing relaxation, leading to a positive affective state and reducing stress and anxiety [42–44], with therapeutic benefits similar to or even more significant than those of traditional programs such as cognitive-behavioral therapy (CBT) interventions (e.g., in vivo exposure and guided imagery) [40, 45]. The visual presentation of relaxing virtual scenarios (e.g., naturalistic environments) can facilitate the practice of individuals and the consequent mastery of relaxation techniques [42–44]. VR is also adopted successfully to deliver biofeedback [46, 47] and mindfulness training [48, 49]. Furthermore, VR represents a potentially helpful tool for psychoeducation [50–52] that refers to interventions aimed at informing people about an illness and in a clear and articulated way, educating individuals on their disease and how to deal with it [53, 54]. Psychoeducation is considered a key part of CBT [55] and can also be an intervention of its own [56, 57].

Interestingly, recent studies investigated the use of VR for diminishing stress and anxiety among healthcare workers during the COVID-19 pandemic. VRelax (i.e., 360° videos of calming natural environments watched via an HMD) effectively reduced stress and induced positive emotions in a sample of ICU nurses [58]. Besides, Tranquil Cinematic-VR simulation (i.e., a 3-min 360° immersive video of a nature scene) lowered stress among frontline healthcare workers in COVID-19 treatment units [59].

Although these findings are promising, significant limitations could impact the efficacy and the adoption of the VR interventions proposed in these studies. The first one is related to their location (i.e., research lab and hospitals) [58, 59]. Healthcare workers may be unable or reluctant to participate in face-to-face sessions to use VR, especially at times of crisis, primarily due to workload and variable schedules [60]. Importantly, recent standalone VR systems have made this technology feasible for everyday in-home use [61]. Thanks to this fact, home-based VR programs represent new promising interventions for remote psychological support [61–63], including relaxation training [64, 65]. The second main limitation of VR interventions tested by previous studies is related to their content [58, 59]. Such programs have only used VR for relaxation [58, 59], while none has also included psychoeducational VR experiences. However, psychoeducation is considered the first step intervention for managing stress and anxiety, with benefits for reducing the intensity of these conditions [66, 67].

Within this context, this randomized controlled trial (RCT) aims to investigate the efficacy and acceptability of a home-based VR training for managing stress and anxiety during the COVID-19 pandemic in a sample of Italian healthcare workers. The intervention will consist of three training sessions performed in a week. In each session, participants will try through an immersive VR standalone system (i.e., Oculus Quest 2), a virtual psychoeducation experience on stress and anxiety (i.e., MIND-VR) [50], followed by a virtual content for relaxation (i.e., The Secret Garden) [64].

### Objectives

The primary objective of this RCT focus on the usefulness of the proposed home-based VR intervention for decreasing stress and anxiety and for enhancing the knowledge about these conditions compared to a control group. The secondary objective is to assess the acceptability of the home-based VR training.

## Methods/design

The study is registered in the ClinicalTrials.gov database (NCT04611399) and has received ethical approval from the Institutional Review Board Committee (IRBC) of the University of Milano-Bicocca, the Foundation IRCCS Carlo Besta Neurological Institute Foundation, and the Istituto Santa Lucia (Roma, Italy). The study will be conducted based on the CONSORT guidelines [68]. If there are any amendments during the trial, they will be registered on ClinicalTrials.gov.

### Design and setting

The study will follow a 2-arm, parallel-group, RCT model. A total of 60 consecutively enrolled individuals (see the “Sample size” section for the power calculation) will be recruited from healthcare workers of three Medical Sites (MS) in Italy: the Foundation IRCCS Carlo Besta Neurological Institute Foundation (Milan, Italy), the Fondazione Europea Ricerca Biomedica (FERB) (Gazzaniga, Italy), and the Istituto Santa Lucia (Roma, Italy). In each MS, 20 participants will be recruited among hospital units that treat or have treated COVID-19 patients (i.e., the emergency department, surgical units, and critical care units).

### Sample size

Power size calculation was performed with GPower 3.1. Considering an anticipated effect size (*f*) of.25, an alpha set at 0.05, 2 groups, and a 0.95 statistical power, the total sample size required is *N* = 54. We, therefore, plan to enroll a total of 60 participants, which should guarantee a proper sample even in case of drop-outs.

### Recruitment

Healthcare workers will be informed about the possibility of participating in the study with oral communication and a formal email from the institutional study referent. They should confirm their intent to join the study by answering that email, and the referent will set an appointment for the screening interview, as described below.

### Eligibility criteria

The participants’ eligibility will be verified through a screening interview with the research psychologist, who will explain the training’s purpose and procedures and conduct an assessment to identify the participants who can enter the study. On this occasion, if the psychologist detects levels and symptoms of stress and/or anxiety of clinical interest, the participant will be sent to the Institutional Psychological Help Desk and excluded from the study.

The study inclusion criteria will be (1) being currently employed as a healthcare worker, (2) maximum age of 65, (3) absence of medical disorders (heart disease or blood pressure, neurological disorders, epilepsy), (4) absence of pharmacotherapy that could interfere with the measured data (psychoactive drugs, anti-hypertensive, anti-depressants), and (5) no significant visual impairment (all with normal visual acuity or corrected to normal).

### Allocation

During the interview, eligible participants who will meet the inclusion criteria described above will receive written information about the procedure and be asked to sign the consent form indicating their willingness to participate in the study. Participants will also be required to fill out baseline questionnaires accessible online. Only qualified individuals who provided informed consent will be randomly assigned to the experimental or control group in a 1:1 manner. Participants will then receive information regarding the allocation result.

### Randomization

The study coordinator will prepare the randomized sequence with a block randomization procedure using a true random number generator: www.randomizer.org. The randomized list will be created using a reasonably sized permuted block method, the block size of which will not be disclosed until the end of this trial to ensure concealment.

### Withdrawn criteria

Participants will be considered withdrawn if any of the following occurs: (1) participant chooses to withdraw from the study at any time, (2) intolerable adverse effects, (3) major violation of the study protocol, and (4) other circumstances that would endanger the health of the subject if he/she were to continue his/her participation in the trial. The date and reason for withdrawal will be documented in a paper-and-pencil case report form (CRF).

### Strategies for monitoring and improving intervention protocol adherence

All participants will be invited to contact the research psychologist as needed. Participants in the experimental group will be provided with a log book to record technical problems, safety issues, and the date and time they used VR during the intervention. Besides, the research psychologist will monitor the intervention adherence using remote access to the online questionnaires dataset. Participants who will not have completed the pre- and post-training session scales on the scheduled dates will receive an email and a text message encouraging them to use VR. Furthermore, the psychologist will send an email and a text message to remind participants to complete the post-intervention and follow-up assessment, respectively, at the end and 1 month after the intervention. Participants in the experimental group will also be reminded to participate in the post-training session. The psychologist will contact via a phone call those who will not fill out online questionnaires at the scheduled times and individuals in the experimental group who will not attend the post-training session.

### Participant timeline

**Fig. 1:**
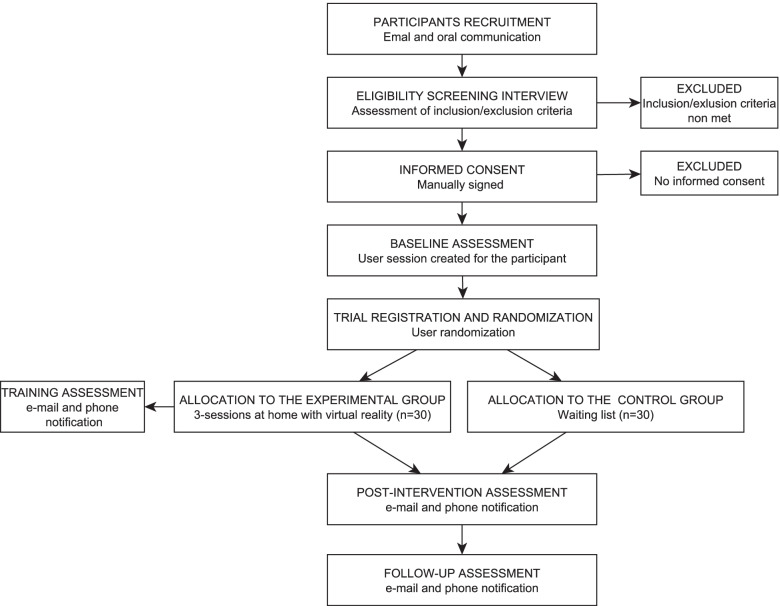
Flow chart of the study design

The trial period is shown in the consolidated standards of reporting trials diagram [68] (Fig. 1) and Standard Protocol Items: Recommendations for Interventional Trials template [69] (Table 2).

**Table 2:** Assessment schedule

	**Study period**					
	**Enrolment**	**Baseline**	**Allocation**	**Intervention**	**Post-intervention**	**Follow-up**
**Timepoint (weeks)**	** − 1**	**0**	**0**	**0**	**1**	**5**
Enrolment:						
Eligibility screen	X					
Informed consent	X					
Allocation			X			
Interventions						
Experimental group				↔		
Control group				↔		
Assessments:						
Demographics		X				
Use of technological solutions and VR		X				
Level of exposure to COVID-19		X				
FCOR		X				
Stress and anxiety management		X				
PSS-10		X			X	X
STAI-Y2		X			X	X
DASS-21		X			X	X
Knowledge of stress and anxiety		X			X	X
STAI-Y1				X		
VAS-A				X		
SUS					X	
SDM					X	
NPS					X	
Difficulties and adverse effects					X	
Interview					X	

*FCOR* Fear of Coronavirus, *PSS-10* Perceived Stress Scale, *STAI-Y2* State-Trait Anxiety Inventory Form Y-2, *DASS-21* The Depression, Anxiety and Stress Scale-21 Items, *STAI-Y1* State-Trait Anxiety Inventory Form Y-1, *VAS-A* Visual Analogue Scale for Anxiety, *SUS* System Usability Score, *SDM* Subjective Difficulty Measure, *NPS* Net Promoter Score

### Protocol

Participants will be randomly assigned to one of the two following conditions:

- Experimental group
- Control group

#### Experimental group

**Fig. 2:**
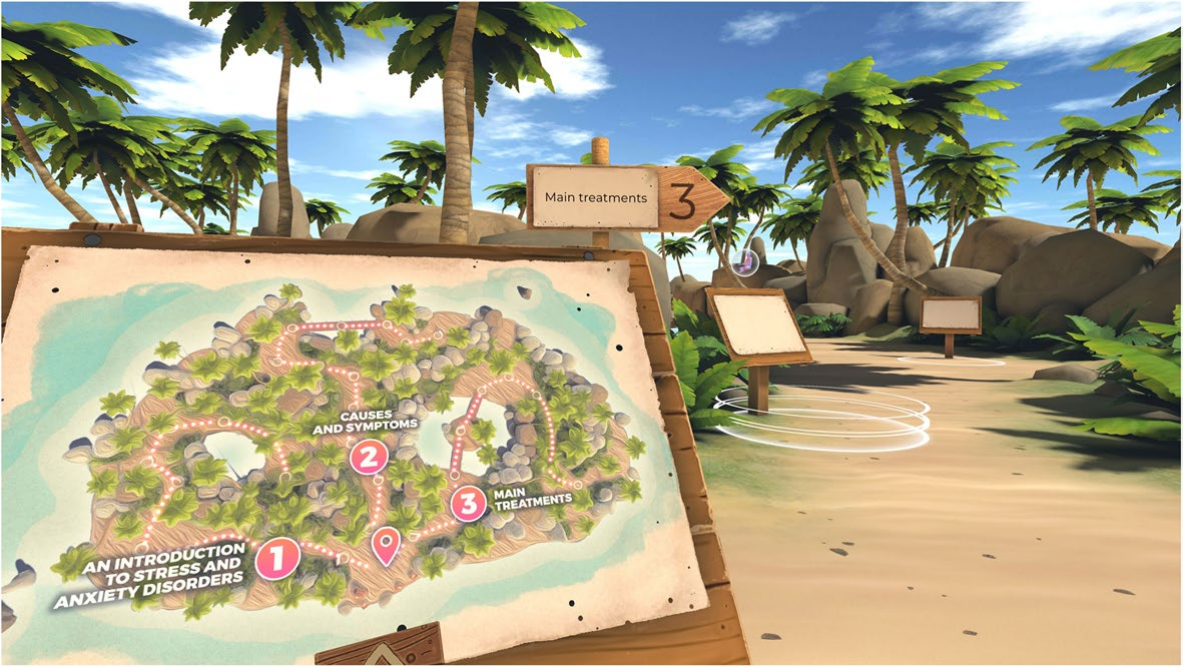
Screenshot of MIND-VR: map of the three paths into which the virtual island is divided: (1) “An introduction on stress and anxiety,” (2) “Causes and symptoms,” and (3) “Main treatments.” Participants in the experimental group will try the first path during the first training session, the second path during the second training session, and the third one during the third training session

Participants in the experimental group will receive the home-based VR intervention. Following a description of the protocol in detail:

- *Intake session (session 1):* after signing the informed consent and completing the baseline questionnaire accessible online (i.e., demographic, ad hoc questionnaire about the use of technological solutions and VR, ad hoc questionnaire on the level of exposure to COVID-19, FCOR, ad hoc questionnaire on stress and anxiety management, PSS-10, STAI-Y2, DASS-21, ad hoc questionnaire on knowledge of stress and anxiety), participants will receive from the research psychologist an explanation about the aims and methods of the home-based VR program. Participants will be asked for their involvement and availability during a week to allow in-home training. The psychologist will remind participants to perform the training three per week, with a distance of 2 days between one session and another. Then, individuals will receive information about the safety and hygiene procedures to prevent COVID-19 infection (see Table 3). The psychologist will give participants the Oculus Quest 2, a consumer-grade standalone VR system that consists of an HMD and two controllers. Individuals will wear the headset and receive a short 15-min training on using the VR system. In particular, they will try the tutorial content available on the Oculus Store. Furthermore, individuals will receive a link to complete the online questionnaires (i.e., STAI-Y1, VAS-A) before and after each session. They will be provided with a log book in which to record technical problems, safety issues, and the date and time they used VR during the intervention. Finally, the research psychologist will schedule the date of the post-training session.
- *Training sessions (sessions 2, 3, and 4):* they will consist of three home sessions of approximately 30 min each, conducted in a week with a distance of 2 days between one session and another. Participants will try for about 15 min one path of the virtual psychoeducational experience MIND-VR in each session (see Fig. 2). Subsequently, they will use for about 10 min the virtual relaxation content The Secret Garden. Individuals will be asked to complete online the VAS-A and the STAI-Y1 at the beginning and the end of each session.
- *Post-training session (session 5):* the research psychologist will meet participants for the post-intervention interview at the end of the training. On this occasion, participants will return the Oculus Quest 2, which will undergo specific procedures to avoid contamination by COVID-19. Participants will be asked to complete within 1 week after completion of the intervention the online questionnaires (i.e., PSS-10, STAI-Y2, DASS-21, ad hoc questionnaire on knowledge of stress and anxiety, SUS, SDM, NPS, ad hoc questionnaire on difficulties and adverse effects).
- *Follow-up session (session 6):* the PSS-10, STAI-Y2, DASS-21, and the ad hoc questionnaire on knowledge of stress and anxiety will be conducted after a 1-month follow-up.

**Table 3:** Safety and hygiene protocol to prevent COVID-19 infection during the intake and the post-training sessions

Safety and hygiene protocol
***Instruct the participant on the safety and hygiene procedure*** •Inform the participant on the importance of the safety and hygiene procedure •During the intake and post-training session, participants will be asked to follow specific precautions described below •Participants with cold-like symptoms will be asked not to go to the experiment and to notify the experimenter
***Follow the following precautions during the intake session*** •To ensure the safety procedures for the prevention of COVID-19 infection, the session will be conducted in a clean and well-ventilated room inside the hospital •Before entering the room, both the experimenter and the participants will have to wash their hands with an alcohol-based disinfectant (at least 70%) •Tell the user to keep at least 1.5 m from the experimenter •The participant will take the headset placed on a table placed about 3 m away from the experimenter and will be explained how to wear it independently and how to use it
***Disinfect the headset after the post-training session*** •As soon as the session is finished, the experimenter will use a detergent with at least 70% alcohol to decontaminate the headset •The cleanable, waterproof face part, keys areas of the headset (i.e., top and bottom, the headbands, buttons), and controllers will be disinfected and allowed to dry on a clean surface •At the end of the procedure, the experimenter will have to disinfect his/her hands again

#### Control group

This group will undergo baseline, post-intervention, and follow-up assessments without undergoing any training during the 1-week intervention period.

### Virtual reality experiences

#### Virtual reality psychoeducational experience on stress and anxiety: MIND-VR

MIND-VR is a VR-based psychoeducational experience on stress and anxiety created by a team from the University of Milano-Bicocca, thanks to the funds raised through a crowdfunding campaign [50]. This virtual experience was developed in collaboration with AnotheReality between July and October 2020 using Unity Engine. MIND-VR runs on one of the currently more widespread, user-friendly, and low-cost HMDs (i.e., Oculus Quest 2), and it can be downloaded for free, both in Italian and English, on the project website (https://mindvr.com/free-download/).

This VR-based psychoeducational experience has been created following the theoretical framework of architectural approaches for the user-centered design (UCD) of virtual environments developed by Gladden [70]. Since the scientific literature suggests that naturalistic virtual environments cause a relaxing effect and an increase in positive emotions [71–73], a small tropical island was chosen as the setting. The virtual island is divided into three areas. Each focused on different aspects related to stress and anxiety and presented to the user through a map at the beginning of the content (Fig. 2). In each area, the users, as if they were inside a museum, can follow a path along which they will find information in text, audio, and image form on the definitions (first path), causes and symptoms (second path), and main treatments (third path) of stress and anxiety. Each of the routes takes about 15 min to be ended.

#### Relaxing virtual experience: The Secret Garden

**Fig. 3:**
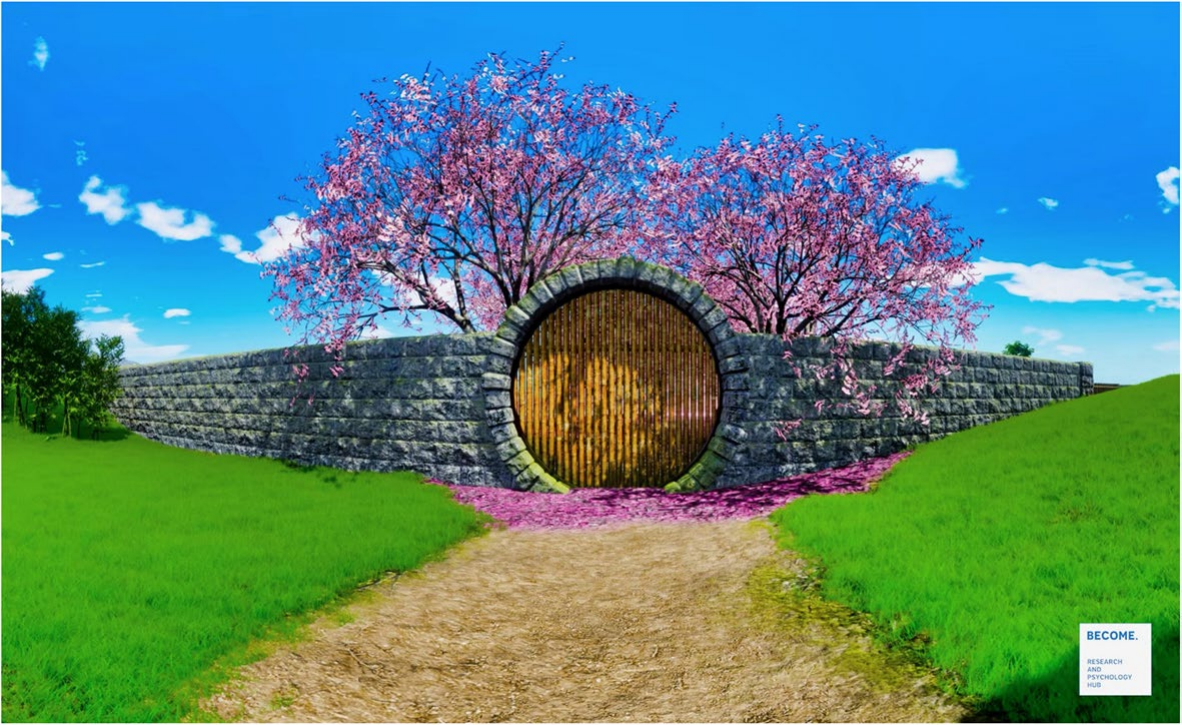
Screenshot of The Secret Garden

The Secret Garden is a 10-min computer graphic 360° video developed by Riva and colleagues [26, 64] for relaxation training and freely available at the website www.covidfeelgood.com. Well-being psychologists have written this experience to mimic the structure and the experience of walking in a Japanese garden [74], providing the visual (i.e., the flow of water) and auditory (i.e., the sound of running water) natural elements available outdoors (Fig. 3).

During The Secret Garden, a slow, calm, clear voice provides a relaxation induction based on Compassion Focused Therapy (CFT) [75, 76]. Specifically, the induction aims to deactivate the human threat protection system and activate the soothing system (with a mindset attended to giving and receiving care, affecting, and nurturance).

#### Hardware

The virtual experiences will be offered through the Oculus Quest 2 (marketed since November 2021 as Meta Quest 2). It was released in October 2020 and is a standalone headset with an internal, Android-based operating system. It is lighter than the first-generation Quest and uses a single fast-switch LCD panel with 1832 × 1920 per eye resolution, and it supports a 90-Hz refresh rate.

### Outcomes

#### Primary outcome measure

At baseline, post-intervention, and follow-up, participants in the experimental and in the control group will complete the following questionnaires that will serve as the primary outcome measures:

- *Perceived Stress Scale* (PSS-10) [77, 78]: the PSS is a 10-item self-reported measure to assess the current stress level using a 5-point Likert scale.
- *State-Trait Anxiety Inventory Form Y-2 (STAI-Y2)* [79, 80]: a validated and widely used measure of trait anxiety. Individuals are asked to specify to which extent, on a 4-point Likert scale (from “not at all” to “very much”), they usually perceive each of the 20 indicated feelings.
- *The Depression, Anxiety and Stress Scale-21 Items (DASS-21)* [81]: is a set of three self-report scales designed to measure depression, anxiety, and stress symptoms. Each of the three scales contains seven items, divided into subscales with similar content. The scores for depression, anxiety, and stress are calculated by adding up the scores for the relevant items.
- Ad hoc* questionnaire on the knowledge of stress and anxiety*: individuals are asked to rate on a 7-point Likert scale (1 = “not at all,” 7 = “very much”) knowledge about the differences between anxiety and stress, causes and symptoms of stress and anxiety, and techniques for the management of these conditions. Besides, participants complete 5 factual questions in multiple-choice format (e.g., “Which is the first phase of the General Adaptation Syndrome?”) and 2 conceptual questions in short-answer format (e.g., “What are the three main categories of stress symptoms?”). The questions are based on the methodology used in a previous study (Parong and Mayer, 2018) (see Table 4).

**Table 4:** The ad hoc questionnaire on the knowledge of stress and anxiety

Questionnaire on the knowledge of stress and anxiety
***Part 1. Rate on a 7-point Likert scale (1* = *“not at all,” 7* = *“very much”) your level of knowledge about*:** • The differences between anxiety and stress • Causes and symptoms of stress and anxiety • Techniques for the management of stress and anxiety
***Part 2. Answer the following questions*:** • What is the first stage of the “general stress adaptation syndrome”? -Resistance phase -Exhaustion phase -Alarm phase -None of these • What is the main difference between stress and anxiety? -Symptoms and duration -Duration and causes -The causes and duration • Which of these is a symptom of social anxiety? -Constant and pervasive anxiety -Avoidance of social situations -Fear of enclosed spaces -None of the above • What is the main difference between acute stress disorder and post-traumatic stress disorder? -Duration -The causes -None of the above • Which of these biofeedback claims is incorrect? -Its primary purpose is to teach how to modify physiological response patterns -It consists of several sessions -Focuses on breathing -None of the above
***Part 3. Open-ended questions*:** • What are the three main categories of stress symptoms? • What are the main techniques for the management of stress and anxiety?

#### Secondary outcome measure

Participants in the experimental group at post-intervention will complete the following questionnaires that will serve as the secondary outcome measures:

- *System Usability Score (SUS) *[82]*:* it consists of 10 items on a 5-point Likert scale (1= “strongly agree,” 5= “strongly disagree”), relating to the possible critical issues encountered during the use of a VR system. A measure is provided on usability aspects (i.e., efficiency, clarity, reliability) and other aspects of the user experience (i.e., originality, stimulation).
- *Subjective Difficulty Measure (SDM):* it consists of a horizontal line 100 mm long, anchored by word descriptors at each end (from “not at all” to “very much”). Users mark on the line the perceived difficulty in using the VR system in general, MIND-VR, and The Secret Garden.
- *Net Promoter Score (NPS) *[83]: it evaluates user satisfaction with a product, in this case: the VR-home-based intervention in general, MIND-V, and The Secret Garden. It requires to indicate on a scale from 0 (“not at all”) to 100 (“very much”) how much they would recommend the products to a family member or friend.
- *Ad hoc questionnaire on difficulties and adverse effects*: individuals are asked to rate on a 7-point Likert scale (1=“not at all,” 7 = “very much”): If they had difficulty following the experimental protocol; if they had nausea, headache, dizziness, and eyestrain while using the VR system in general; if they had nausea, headache, dizziness, and eyestrain while using MIND-VR; if they had nausea, headache, dizziness, and eyestrain experienced while using The Secret Garden.

Furthermore, adherence to study protocols will be assessed through data on VR usage recorded on the log book, training sessions completed, assessments attended, and drop-outs. Data recorded by participants on the log book regarding technical problems and safety issues will serve to evaluate the usability of the VR system.

Finally, during the post-training session, participants of the experimental group will be interviewed for about 15 min. The questions will be based on the methodology used in a recent study [51]: Were there any features that you especially liked in using the home-based VR training? Were there any features you did not like or found useless? Have you ever had to stop using VR, and if so, why? Have you learned anything using MIND-VR and The Secret Garden? Were there any features of the VR experiences more valuable than others? Would you like to have more sessions with this type of training? Do you have any questions or would like to add anything?

#### Other measures

At baseline, participants in the experimental and in the control group will complete:

- *Demographic*: genre, age, years of education, profession, hospital, work department, years of professional seniority
- *Ad hoc questionnaire on the use of technological solutions and VR*: individuals will be asked to indicate on a 7-point Likert scale (from “not at all” to “very”): the level of use in general of technological devices, the general level of acceptance of the technology, the level of knowledge of VR, and the level of experience with VR systems.
- *Ad hoc questionnaire on the level of exposure to COVID-19: *individuals are asked to answer the following questions (“yes”/“no”): Have you ever been caring for COVID-19 patients? Have you ever had family members or friends who became ill with COVID-19? Have you ever had family members or friends who passed away due to COVID-19? Have you ever had any laboratory tests (e.g., serological test, swab) for COVID-19? If you answered “Yes” to the previous question, how many times have you had a laboratory test for COVID-19? Have you ever tested positive for COVID-19? If you answered “Yes,” please indicate how many weeks you have been affected by COVID-19 and the level of severity of symptoms (from 1 “very mild” to 7 “very severe”).
- *Fear Of Coronavirus (FCOR) *[84]: it consists of a series of statements to measure the level of fear toward the COVID-19 pandemic. The questionnaire is composed of eight items that explore different components of fear, such as the personal experience of concern regarding the current situation, avoidance behaviors, and attention bias. Each statement is evaluated on a 5-point Likert scale.
- *Ad hoc questionnaire on stress and anxiety management*: 10-item scale developed to assess the use of stress and anxiety management programs and their perceived usefulness (see Table 5).

**Table 5:** The ad hoc questionnaire on stress and anxiety management

Questionnaire on stress and anxiety management
**Part 1. Please indicate if (“yes”/ “no”):** • Have you ever followed psychotherapy? • Have you ever followed stress and anxiety management programs? • Since the COVID-19 outbreak, have you ever used remote stress and anxiety management programs (telephone or video)? • Since the COVID-19 outbreak, have you ever used face-to-face stress and anxiety management programs (single or group sessions)?
**Part 2. Rate on a 7-point Likert scale (1 = “not at all,” 7 = “very much”):** • How important in general do you think to have psychological support for stress management and anxiety concerning your profession? • How important do you think it is to have psychological support for managing stress and anxiety in times of emergency, such as those experienced during the COVID-19 crisis? • How important do you feel to follow a stress and anxiety management program at this time in your life? • Have you ever attended meetings about stress and anxiety? • Have you ever searched the Internet for information on stress and anxiety? • Have you ever read books on stress and anxiety?

Besides, with the aim of measuring changes in the affective states of individuals during the intervention, participants of the experimental group will be asked to fill before and after each of the training session the following self-report questionnaires:

- *State-Trait Anxiety Inventory Form Y-1* (STAI-Y1) [79, 80]: STAY-Y1 addresses state anxiety, which could be defined as a temporary emotional condition characterized by apprehension, tension, and fear about a particular situation or activity. This inventory consists of 20 items on a 4–4-point Likert scale, like the STAI-Y2.
- *Visual Analogue Scale for Anxiety* (VAS-A) [85, 86] is an instrument that measures state anxiety across a continuum. It is a horizontal line, 100 mm in length, anchored by word descriptors at each end (“no anxiety”; “very severe anxiety”). Individuals mark on the line the point that they feel represents their perception of their current state. The VAS-A score is determined by measuring in mm from the left-hand end of the line to the point that the person marks.

### Data management

Epidemiological and clinical data from each participant will be collected on the CRF by the research psychologist and recorded online all over the study by the study coordinator. Audit trails will be integrated for tracking data entry correction and import procedures for data monitoring. All data gathered during the study will be stored anonymously in a web Central Database Repository. Security and privacy issues will be taken care of according to the individual situation and the participant’s consent. A unique participant number will be assigned to every operator consisting of name and surname first letters and a consecutive number. This unique code will identify all participant-specific data (e.g., epidemiological and clinical study data). Any adverse event will be documented on the CRF. Data access and storage will follow the data security concept of the MS, including password-protected access to all computers and folders. Data will be stored for up to 5 years after the study ends.

### Data analysis

Analysis of variance will be used to evaluate baseline characteristics of the two groups involved in the study, the overall significance of improvement across primary outcome measures, and drop-out versus maintainers. Categorical variables will be compared using Fisher or chi-square tests and continuous variables using *t*-test or Mann–Whitney tests, as appropriate. To assess the effectiveness of the intervention, groups will be compared with a 2 × 2 repeated measure mixed ANOVA for the pre- and post-primary outcome measures (factor Group X factor Time: pre and post). The analysis will be performed for the PSS, STAY-Y2, and DASS-21. A repeated-measures ANOVA (factor Group × factor Time: day of the week) will compare treatment effects within the 1-week intervention for the STAY-Y2 and the VAS-A. Tests of statistical significance and confidence intervals will be two-sided. A *p* < 0.05 will be considered statistically significant. Descriptive methods will be used to report the usability of the intervention for the secondary outcome intervention (i.e., SUS, SDM, NPS, ad hoc questionnaire on difficulties and adverse effects). Data analyses will be carried out through SPSS software.

Recordings from the post-intervention interviews will be transcribed. Two researchers will read the interview transcripts carefully several times to have a sense of the whole data, identifying emerging themes and categories [87]. A codebook will be created and modified until the final set of codes is obtained and reached to the data saturation point when there is no emergence of other new themes. Qualitative data analysis will be conducted using a thematic content analysis approach following a systematic process. Codes will be applied to the transcripts and converted into categories to represent the main themes arising from the data [88]. Triangulation of themes and concepts will be used to compare further and contrast the data from the different participating groups. Codes will be applied to the transcripts and converted into categories to represent the main themes arising from the data [88].

### Data monitoring

This RCT study has been designed for conciseness and minimizing risk. Therefore, no formal data monitoring committee has been organized. In addition, no interim analysis of the intervention’s impact has been planned at this stage.

### Ethics

During the post-training session, participants of the experimental group will have a debriefing with the research psychologist. Should any participants experience adverse emotional reactions after the session, they will be addressed to appropriate psychological support, and the possibility of withdrawal from the study will be considered. These events will be recorded. Specifically, the time point at which the adverse events occurred, the motivation, and the events’ features (which emotions and with which intensity, which psychophysiological perceptions) will be collected and recorded on the CRF. Any adverse event will be discussed within the project team and assessed individually to identify any variable needing revision within the virtual content. If any substantial protocol modifications are needed because of organizational and/or clinical reasons, the study will be promptly suspended.

### Auditing

No audit has been planned at this time.

### Protocol amendments

Protocol amendments, including informed consent for participants, will be sent to the ethical committees for evaluation. Once the amendments are approved, a modified informed consent form will be given to participants and signed.

### Access to data

Prior to the publication of major results, only the study coordinator and individuals approved by the study coordinator will have access to the complete dataset. They will address problems related to the data and finalize a dataset for statistical analysis. After publication, the dataset created during this study will be available from the study coordinator on reasonable request.

### Ancillary and post-trial care

The study does not involve any physical, psychological, or social risks. Participants will be able to contact the research psychologist during the trial period. The study could be stopped if any adverse effects related to the use of VR (i.e., nausea, vertigo) will be reported by the participant during the intervention.

### Dissemination plans

The study outputs and methodology will be disseminated worldwide to professionals and researchers to promote the exploitation of project findings in future confirmatory studies and in recommendations for the management of healthcare professionals’ mental health. The use of project findings in further studies and replication in other contexts will provide external validity to the results and favor a potential standardization impact.

### Discussion

The main aim of this RCT study is to evaluate the usefulness and the acceptability of a brief home-based VR training for the management of stress and anxiety in healthcare workers. Since stress and anxiety have increased after the outbreak of the COVID-19 crisis [4–7] and given that these conditions can have harmful effects on both health and work performance [17, 18, 20, 21], the study’s findings may offer valuable insights for developing innovative psychological interventions to help hospital staff.

If our brief home-based VR training will produce a significant decrease in stress and anxiety and an increase in the knowledge of these conditions, it could become an empirically assessed viable option for protecting the mental health of healthcare workers both during the pandemic and once the emergency ends. Furthermore, it could easily be adapted to other categories of people who need support in managing stress and anxiety. Indeed, the proposed intervention is characterized by the fact that it uses free and ready-to-use virtual experiences, available in different languages, including English (e.g., MIND-VR and The Secret Graden). For example, the proposed VR intervention can be used within programs to manage stress and anxiety for all those people forced into isolation or an extended hospital stay due to COVID-19 infection or other physical disorders. Also, since Summer 2021, this type of approach has been tested in collaboration with a Canadian university and an Italian hospital, respectively, to train future doctors and nurses and provide psychological support to families of patients with dementia.

In terms of format and content, the brief home-based VR training proposed in this study has many advantages over other VR-based interventions previously tested for managing stress and anxiety of healthcare workers [58, 59]. First of all, to the best of our knowledge, this is the first study to propose the use of VR at home and not in a hospital or laboratory. In our study, healthcare workers will access the VR training directly in their homes and at times that are most convenient for them. Data collected by this study will offer helpful insights on the efficacy and acceptability of VR-based training, which represents an interesting new way to offer remote pisco support and which could facilitate treatment adherence compared to face-to-face VR interventions [61–63]. Importantly, in this study, we will use VR standalone systems, which, compared to other types of immersive systems that are easy to use even at home, such as mobile ones, offer a higher quality experience with more possibilities for interaction. Secondly, unlike previous research [58, 59], the training proposed by this study/protocol involves using VR not only for relaxation but also for psychoeducation on stress and anxiety. As suggested by the literature, this fact appears essential as it could increase treatment adherence and lower self-stigma [66, 67].

This study protocol has some limitations. First of all, a third control arm (i.e., treatment as usual) and the physiological monitoring of individuals will not be possible due to the limitation of cost and feasibility. Secondly, it will not be possible to guarantee that the participants themselves performed the training session or that another individual had replaced them. To date, this drawback is common with most (if not all) home-based training programs. Finally, as healthcare workers who volunteered to participate may have been more stressed and or receptive to a VR intervention, the responses may be susceptible to selection bias.

To summarize, if the data collected will give evidence of the efficacy and acceptability of our brief home-based VR training for managing stress and anxiety, the proposed approach can be implemented quickly and cheaply as add-on treatment or continued care to enhance the effectiveness of current evidence-based interventions for healthcare workers. Besides, the proposed training might be easily adapted for other categories of people who need support in managing stress and anxiety.

### Trial status

Patient recruitment was ongoing at the time of manuscript submission. Data collection will continue at least until March 2023.

## Data Availability

The datasets of the study are available on request from the authors.

## Abbreviations

2D: Two-dimensional
3D: Three-dimensional
CAVE: C-Automatic Virtual Environment
CBT: Cognitive-behavioral therapy
CFT: Compassion Focused Therapy
COVID-19: Novel coronavirus disease
CRF: Case report form
DASS-21: Depression, Anxiety, and Stress Scale-21 Items
FCOR: Fear of coronavirus
FERB: Fondazione Europea Ricerca Biomedica
HMDs: Head-mounted displays
IRBC: Institutional Review Board Committee
MANOVA: Multivariate analysis of variance
NPS: Net Promoter Score
PSS-10: Perceived Stress Scale
PTSD: Post-traumatic stress disorder
RCT: Randomized controlled trial
SDM: Subjective Difficulty Measure
STAI-Y1: State-Trait Anxiety Inventory Form Y-1
STAI-Y2: State-Trait Anxiety Inventory Form Y-2
SUS: System Usability Score
UCD: User-centered design
VAS-A: Visual Analogue Scale for Anxiety
VR: Virtual reality
WHO: World Health Organization
